# Supplementary material for: Oral Citrate Supplementation Mitigates Age‐Associated Pathologic Intervertebral Disc Calcification in LG/J Mice
Source: Aging Cell. 2025 Feb 11;24(5):e14504. doi: 10.1111/acel.14504 (PMC12073913; doi:10.1111/acel.14504)
Supplement: Supplementary file 1 — Figure S1. Figure S2. Figure S3. Figure S4. Figure S5. Figure S6. Figure S7. Figure S8. Figure S9. [file ACEL-24-e14504-s002.pdf]

Supplementary Figure 1

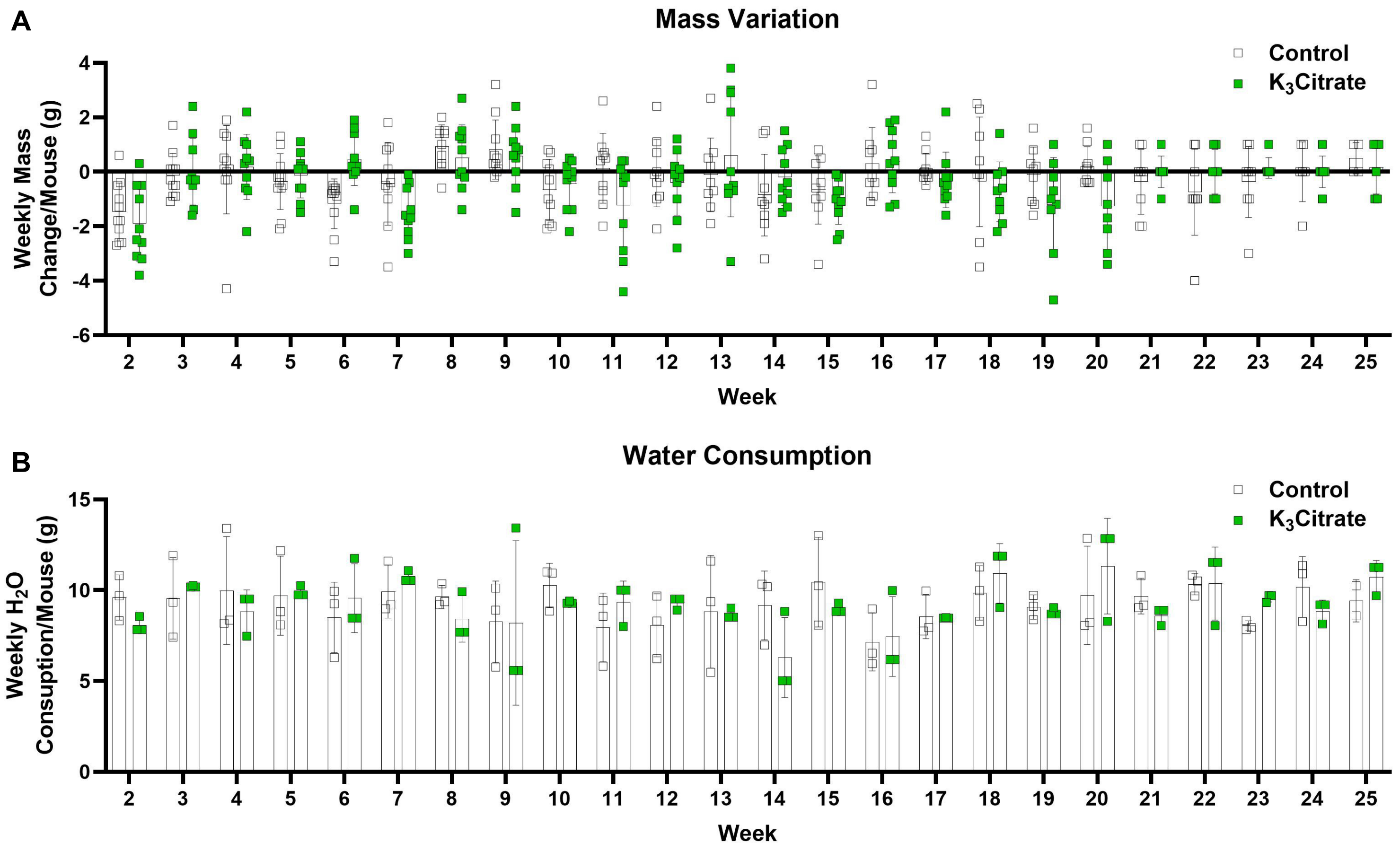

Supplementary Figure 1. K_3Citrate does not impact mass fluctuations of water consumptions in aging LG/J mice. (A) Quantifications of weekly mass changes in control and K_3Citrate mice showed no impact of the treatment on mass fluctuations during aging. **(B)** Weekly water consumption was also unchanged by K_3Citrate supplementation to the water. (Control mice: n=6-11 mice (variation based on weekly mouse survival); K_3Citrate mice: n=6-10 mice (variation based on weekly mouse survival) Data are shown as mean ± SD. Significance was determined to be insignificant between treatment cohorts at each time point using an unpaired t-test or Mann-Whitney test, as appropriate.

Supplementary Figure 2

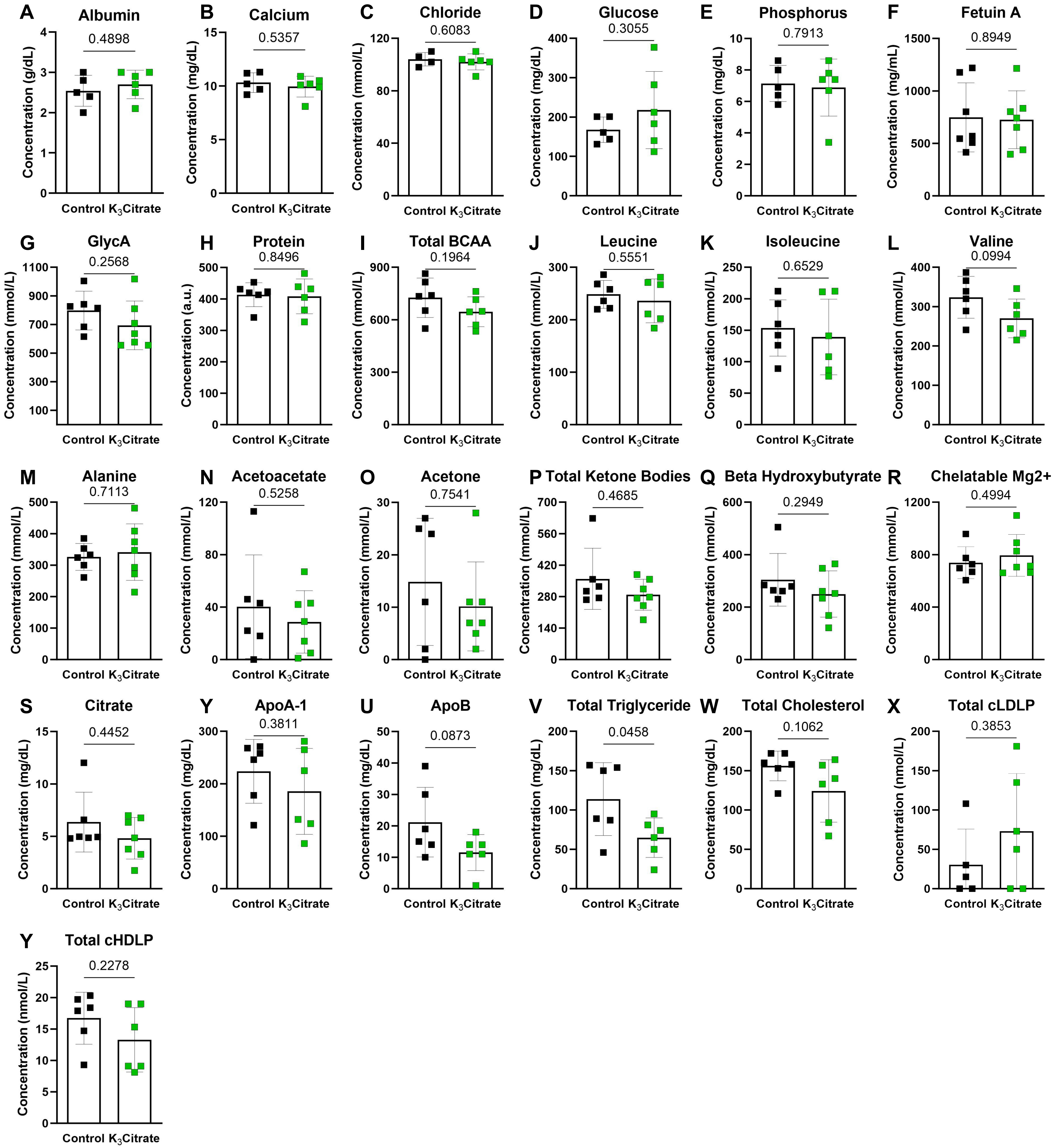

Supplementary Figure 2. Plasma chemistry is stable with K_3Citrate supplementation in LG/J mice. (A) Albumin, **(B)** Calcium, **(C)** Chloride, **(D)** Glucose, and **(E)** Phosphorus were analyzed using an IDEXX BioAnalytics custom blood chemistry panel (Control: n=5 mice (1F, 4M); K_3Citrate: n=6 mice (2F, 4M)). **(F)** Fetuin-A, an inhibitor of mineralization, showed no differences across groups (Control: n=7 mice (2F, 5M); K_3Citrate: n=7 mice (3F, 4M)). **(G)** GlycA, **(H)** Protein, **(I)** total branched chained amino acids (BCAA), **(J)** Leucine, **(K)** Isoleucine, **(L)** Valine, **(M)** Alanine, **(N)** Acetoacetate, **(O)** Acetone, **(P)** Total Ketone Bodies, **(Q)** Beta Hydroxybutyrate, **(R)** Chelatable Mg2+, **(S)** Citrate, **(T)** ApoA-1, **(U)** ApoB, **(V)** Total Trigliyceride, **(W)** Total Cholesterol, **(X)** total calibrated low-density lipoprotein particle (cLDLP), and **(Y)** total calibrated high-density lipoprotein particle (cHDLP) were measured using NMR at LabCorp Global Research Services, and showed no changes with K_3Citrate supplementation (Control: n=6 mice (1F, 5M); K_3Citrate: n=7 mice (3F, 4M)) . Sample size varied between assays based on the volume of plasma required and the volume of plasma collected from each mouse. Significance was determined using an unpaired t-test or Mann-Whitney test, as appropriate.

Supplementary Figure 3

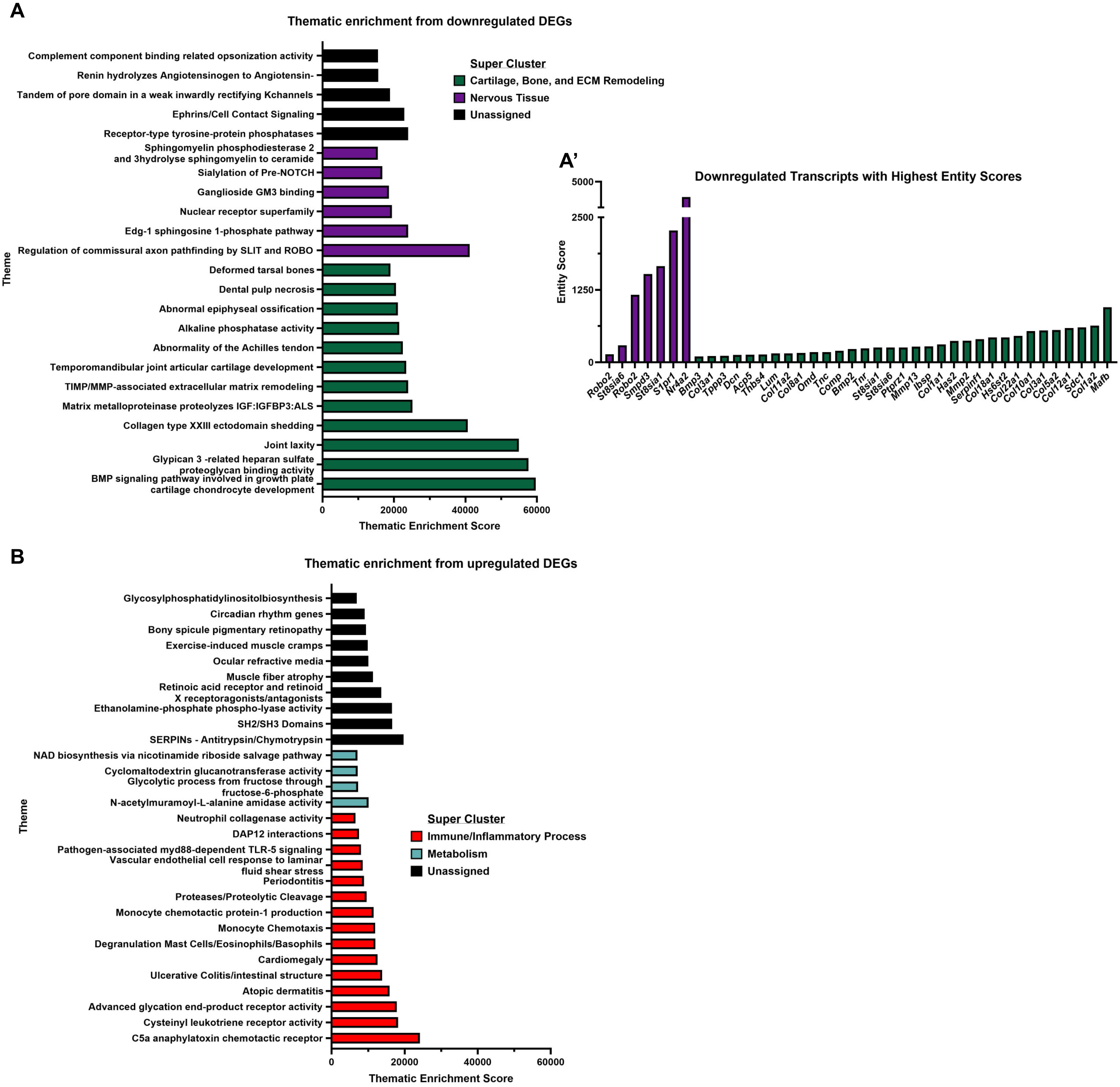

Supplementary Figure 3. Analysis of upregulated DEGs and most enriched downregulated transcripts. (A) RNA-sequencing analysis shows downregulated DEGs have high thematic enrichment. **(A')** Transcripts within each super cluster that have the highest entity scores (cutoff of 100). **(B)** RNA-sequencing analysis shows upregulated DEGs have high thematic enrichment. (Control: n=4 mice (1F, 3M); K_3Citrate: n=7 mice (2F, 2M))

Supplementary Figure 4

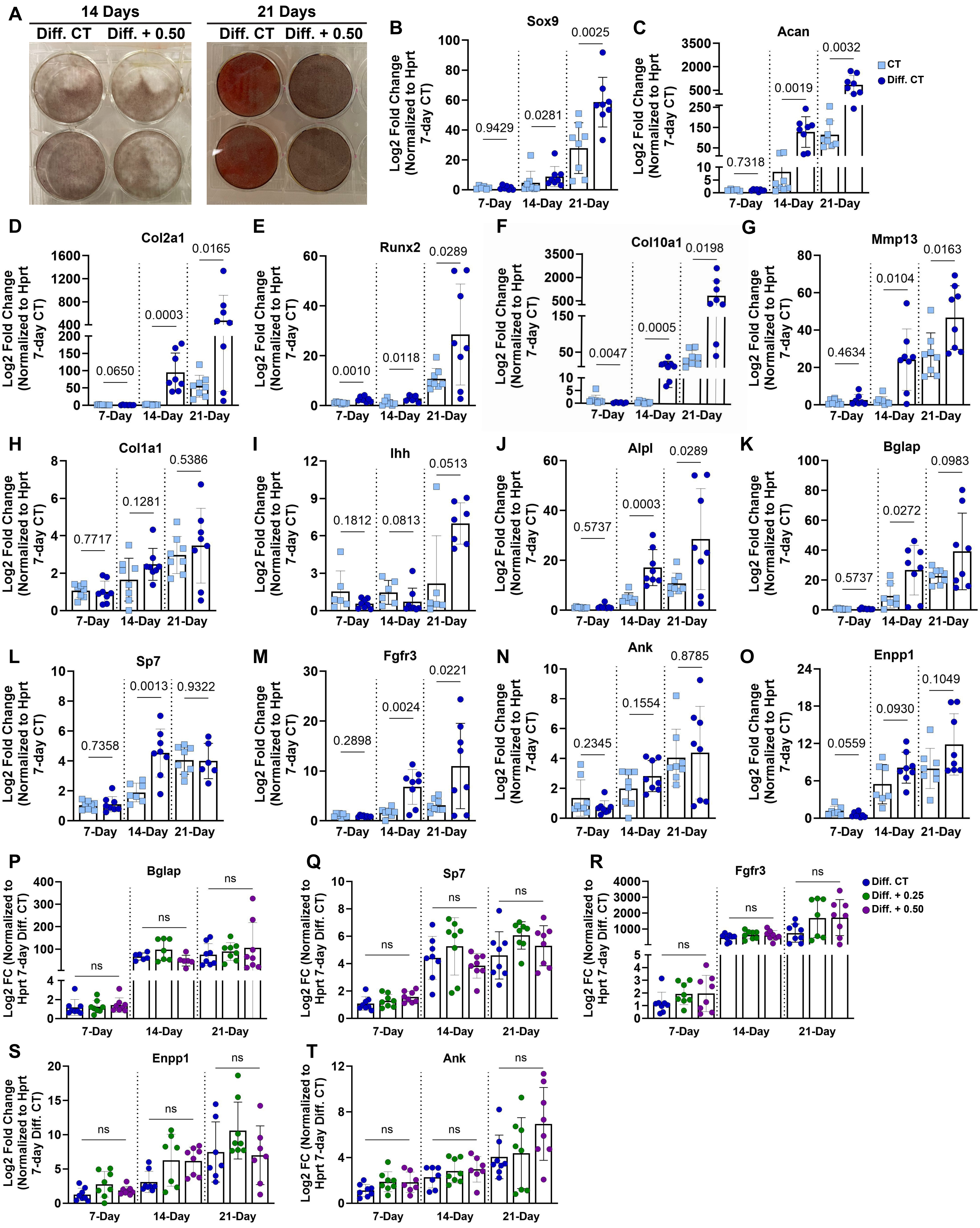

Supplementary Figure 4. Representative Alizarin Red staining, validation of accelerated differentiation protocol in ATDC5 cells, and evidence citrate does not impact cell differentiation. (A) Representative images of Alizarin Red staining in differentiated control (Diff. CT) and differentiated ATDC5 cells treated with 0.50 mM K_3Citrate (Diff. + 0.50). CT and Diff. CT treatment groups were compared for all chondrogenic markers assessed to verify accelerated differentiation in ATDC5 cells receiving β-glycerophosphate and ascorbic acid: **(B)** Sox9, **(C)** Acan, **(D)** Col2a1, **(E)** Runx2, **(F)** Col10a1, **(G)** Mmp13, **(H)** Col1a1, **(I)** Ihh, **(J)** Alpl, **(K)** Bglap, **(L)** Sp7, and **(M)** Fgfr3, **(N)** Ank, and **(O)** Enpp1. (n=8 sets/timepoint, 2 averaged replicates/set) Data are shown as mean ± SD. Significance was determined using an unpaired t-test or Mann-Whitney test, as appropriate. Expression of **(P)** Bglap, **(Q)** Sp7, **(R)** Fgfr3, **(S)** Ank, and **(T)** Enpp1 were unchanged with K_3Citrate supplementation. (n=8 sets/timepoint, 2 averaged replicates/set) Data are shown as mean ± SD. Significance was determined using an ANOVA or Kruskal-Wallis test, as appropriate.

Supplementary Figure 5

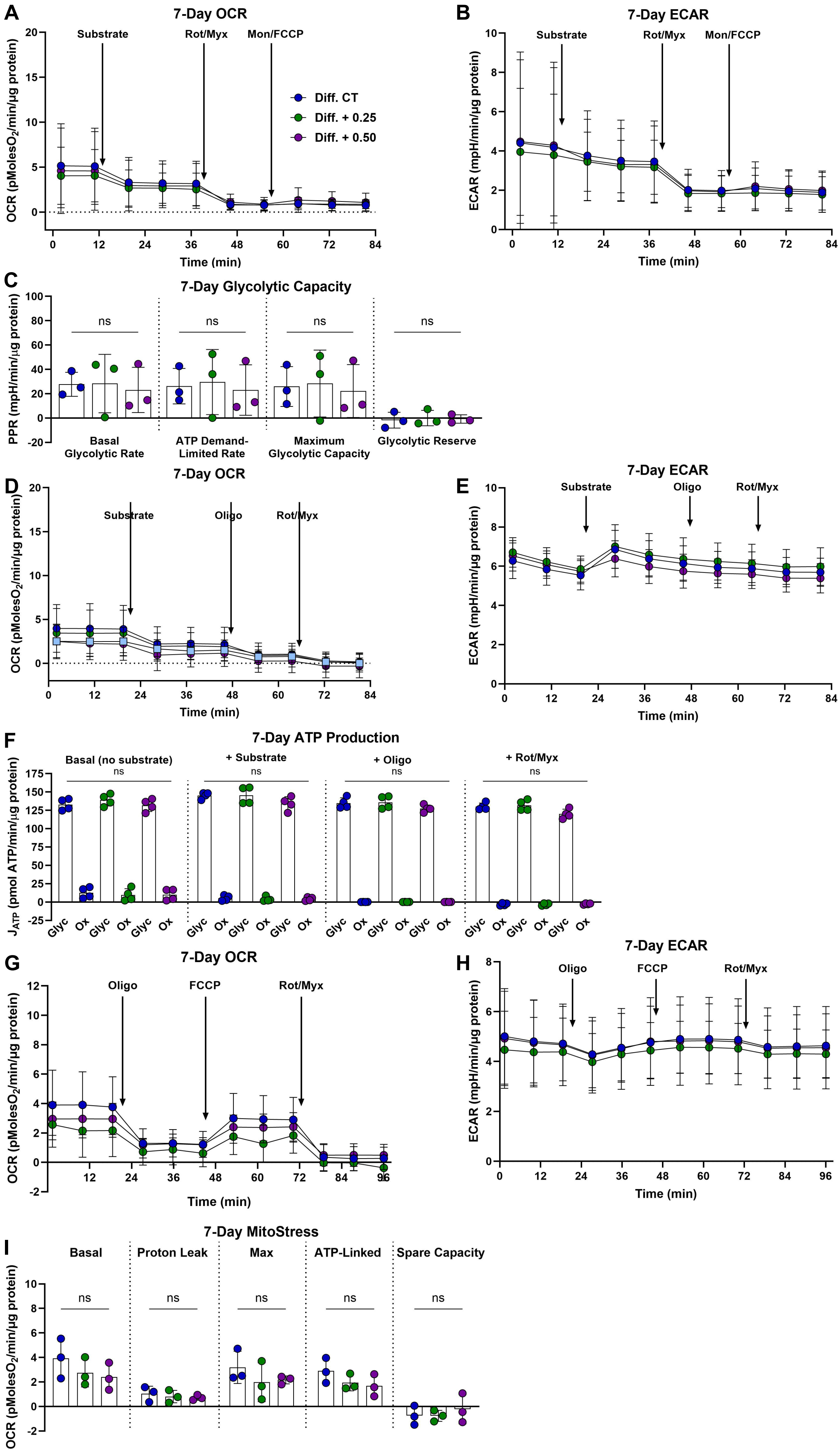

Supplementary 5. K_3Citrate supplementation does not impact glycolytic or oxidative metabolism in ATDC5 cells cultured for 7 days. (A) OCR and **(B)** ECAR traces for ATDC5 cells cultured with or without K_3Citrate **(C)** to evaluate glycolytic capacity and glycolytic reserve. **(D)** OCR and **(E)** ECAR traces for ATDC5 cells cultured with or without K_3Citrate **(F)** to evaluate glycolytic and oxidative ATP production rates. **(G)** OCR and **(H)** ECAR traces for ATDC5 cells cultured with or without K_3Citrate **(I)** for the classical Mito Stress test to evaluate key parameters of mitochondrial function. (n = 3 sets, 3-4 replicates/set) Data are shown as mean ± SD. Significance was determined using an ANOVA or Kruskal-Wallis test, as appropriate.

Supplementary Figure 6

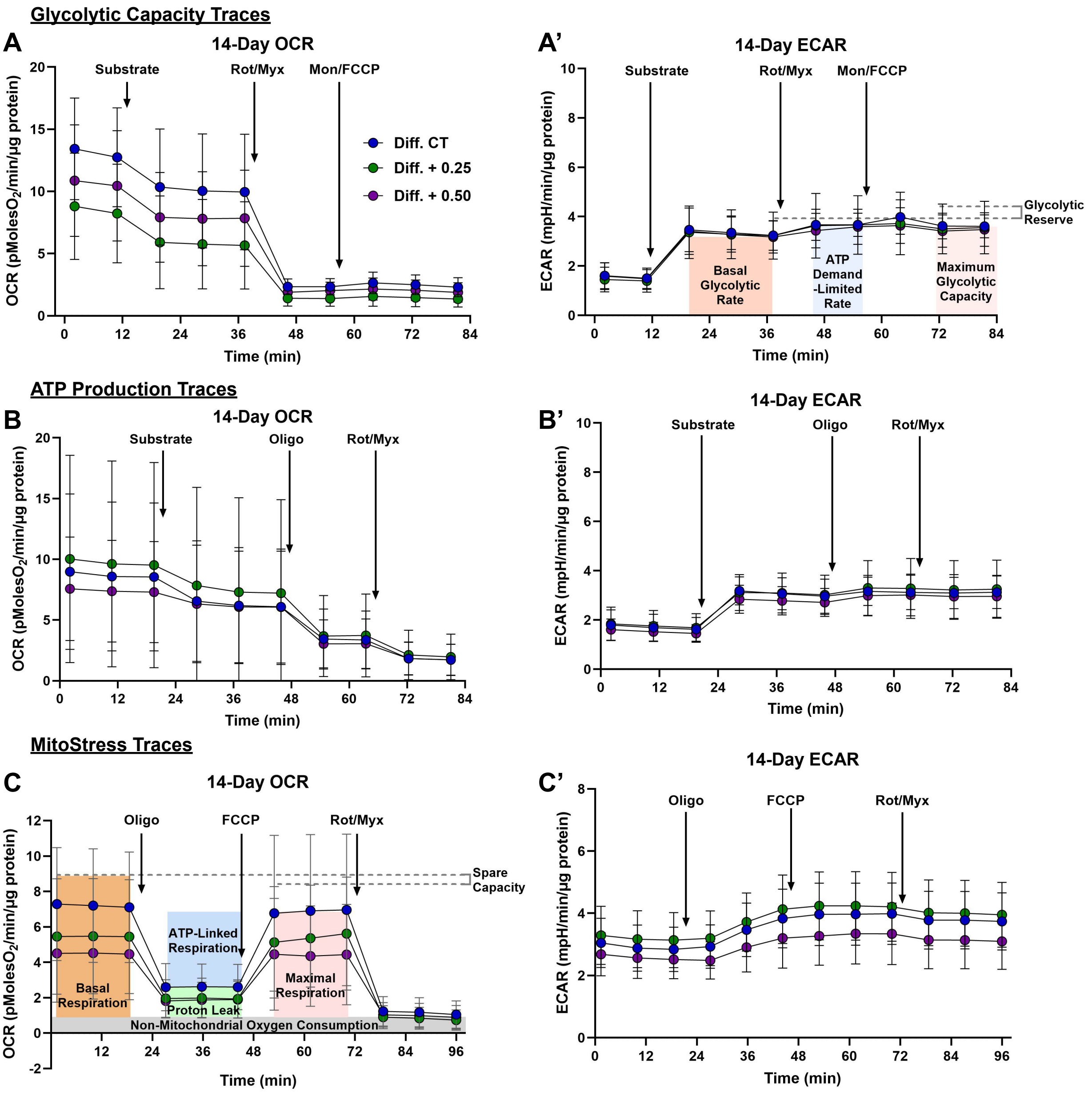

Supplementary Figure 6. K_3Citrate supplementation does not impact glycolytic or oxidative metabolism in ATDC5 cells cultured for 14 days. (A) OCR and **(A')** ECAR traces for ATDC5 cells cultured with or without K_3Citrate evaluate glycolytic capacity and glycolytic reserve. **(B)** OCR and **(B')** ECAR traces for ATDC5 cells cultured with or without K_3Citrate to evaluate glycolytic and oxidative ATP production rates. **(C)** OCR and **(C')** ECAR traces for ATDC5 cells cultured with or without K_3Citrate for the classical Mito Stress test to evaluate key parameters of mitochondrial function. (n = 3 sets, 3-4 replicates/set) Data are shown as mean ± SD. Significance was determined using an ANOVA or Kruskal-Wallis test, as appropriate.

Supplementary Figure 7

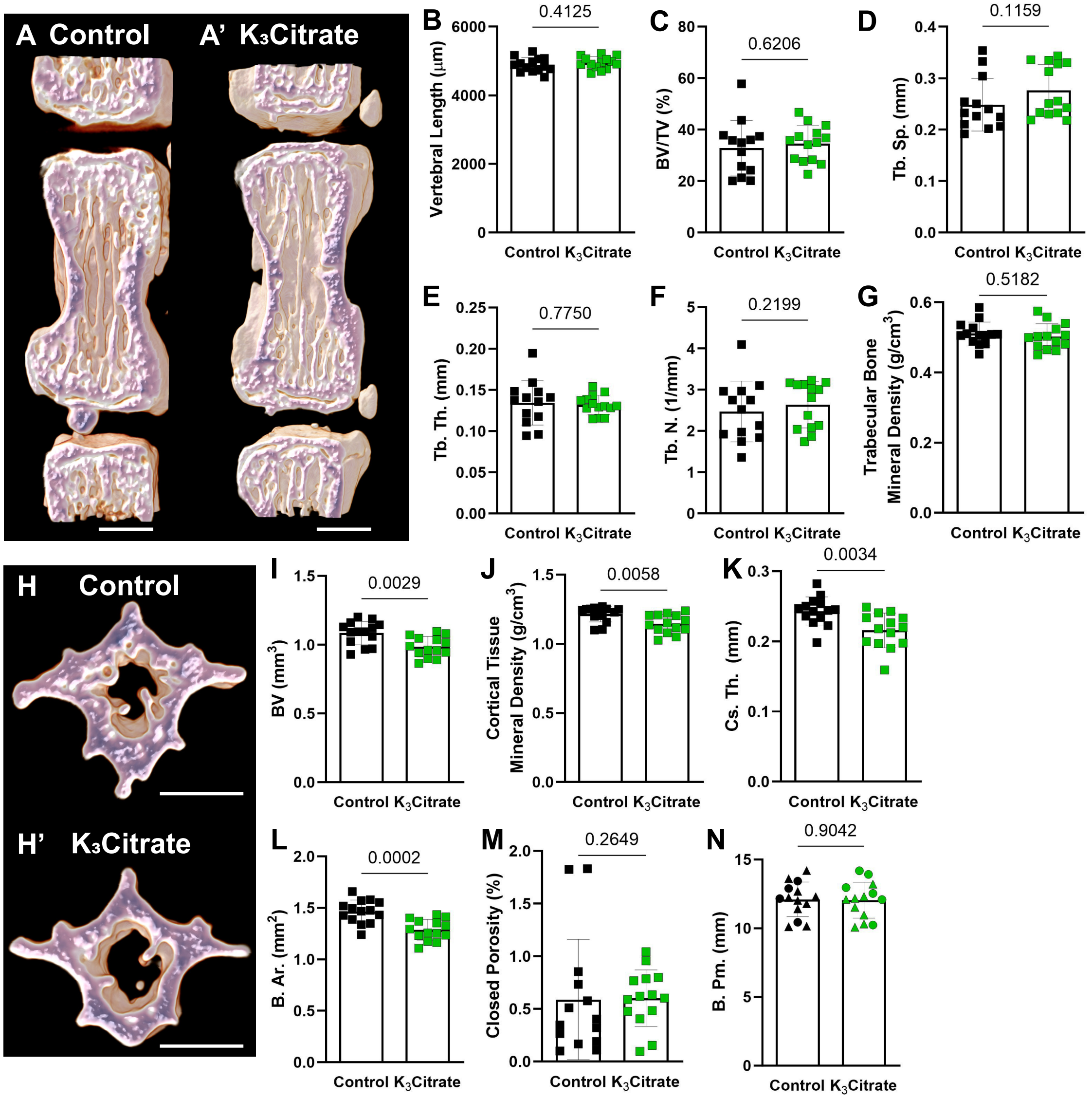

Supplementary Figure 7. K_3Citrate supplementation minimally impacts the caudal vertebrae in LG/J mice. (A-A') Representative μCT reconstructions of the hemi-section caudal motion segments., and **(B)** vertebral length was unchanged. Trabecular properties of **(C)** bone volume fraction (BV/TV), **(D)** trabecular separation (Tb. Sp.), **(E)** trabecular number (Tb. N.), **(F)** trabecular thickness (Tb. Th.), and **(G)** trabecular bone mineral density were unchanged in K_3Citrate mice. **(H-H')** Representative μCT reconstructions central cross sections of the caudal vertebrae. Analysis of the cortical properties **(I)** bone volume (BV), **(J)** cortical tissue mineral density, **(K)** cross sectional thickness (Cs. Th.), **(L)** mean cross-sectional bone area (B. Ar.), **(M)** closed porosity, and **(N)** bone perimeter (B. Pm.) revealed cortical thinning of K_3Citrate caudal vertebrae. (Control mice: n=7 mice (2F, 5M); K_3Citrate mice: n=7 mice (3F, 4M); 2 vertebrae/mouse; 28 discs, 14 vertebrae/treatment) Data are shown as mean ± SD. Significance was determined using an unpaired t-test or Mann-Whitney test, as appropriate.

Supplementary Figure 8

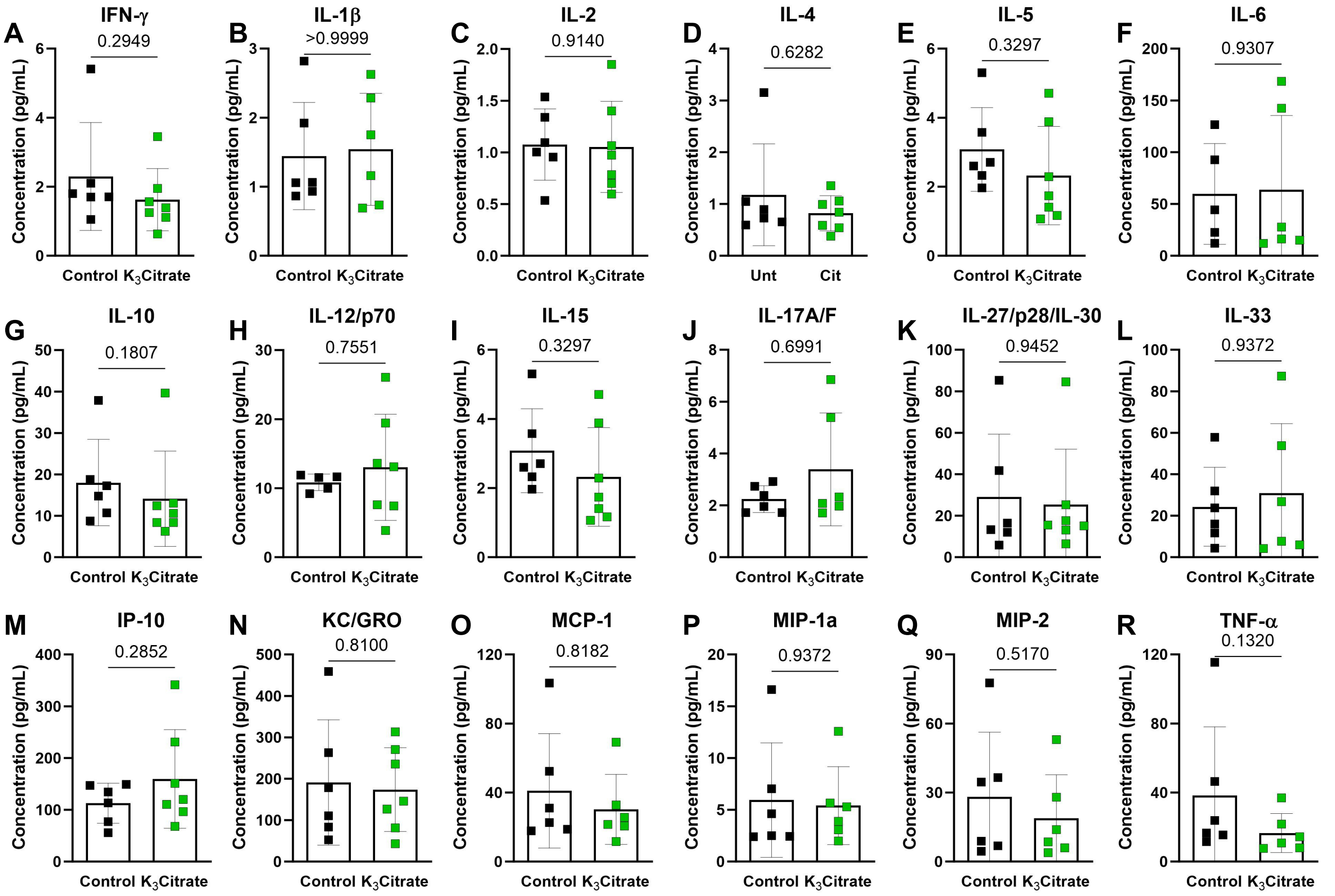

Supplementary Figure 8. Plasma analysis does not indicate attenuation of systemic inflammation. Multiplex assay analysis showed no significant changes in the plasma concentrations of **(A)** IFN-γ, **(B)** IL-1β, **(C)** IL-2, **(D)** IL-4, **(E)** IL-5, **(F)** IL-6, **(G)** IL-10, **(H)** IL-12/p70, **(I)** IL-15, **(J)** IL-17A/F, **(K)** IL-27/p28/IL-30, **(L)** IL-33, **(M)** IP-10, **(N)** KC/GRO, **(O)** MCP-1, **(P)** MIP-1α, **(Q)** MIP-2, **(R)** TNF-α (Control: n=6 mice (1F, 5M); K_3Citrate: n=7 mice (3F, 4M)). Data are shown as mean ± SD. Significance was determined using an unpaired t-test or Mann-Whitney test, as appropriate.

Supplementary Figure 9

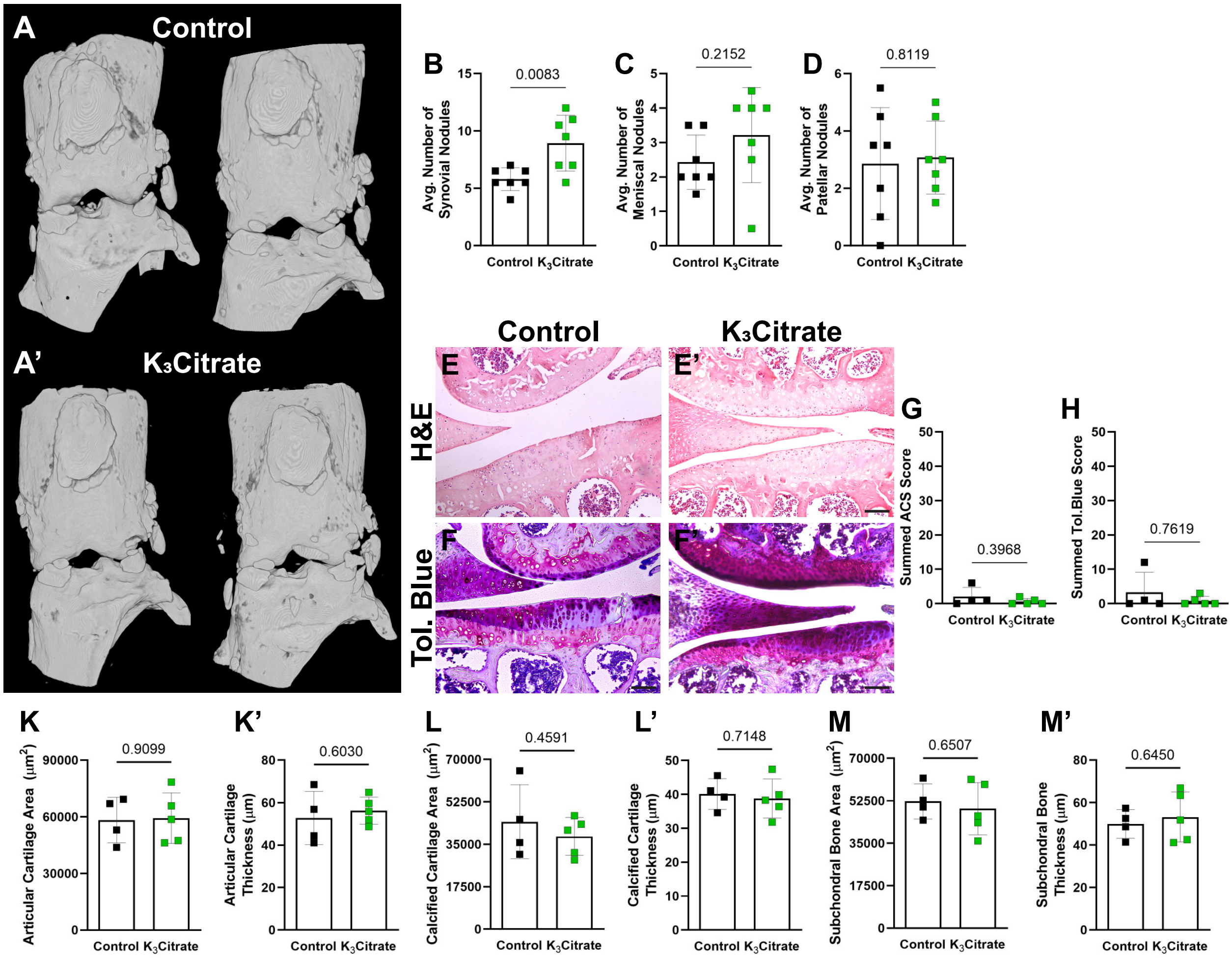

Supplementary Figure 9. K_3Citrate alters knee calcification without impact on cartilage and bone morphology. (A-A') Representative μCT reconstructions of the knees of control and K_3Citrate mice. Quantification of calcification nodules in the **(B)** synovium, **(C)** meniscus, and **(D)** patellar. **(E-E')** Representative hematoxylin and eosin (H&E) staining and (**F-F')** toluidine (Tol.) blue staining of the lateral tibial plateau. Summed **(G)** ACS score, **(H)** toluidine blue score, **(I)** osteophyte score, and **(J)** synovial hyperplasia score show no difference between cohorts. Additionally, K_3Citrate supplementation did not result in changes to the area or thickness of **(K-K')** articular cartilage, **(L-L')** calcified cartilage, or **(M-M')** subchondral bone. (Control: n=4 mice (1F, 3M); K_3Citrate: n=5 mice (3F, 2M)). Data are shown as mean ± SD. Significance was determined using an unpaired t-test or Mann-Whitney test, as appropriate.
